# Where Do Milk Microbes Originate? Traceability of Microbial Community Structure in Raw Milk

**DOI:** 10.3390/foods14091490

**Published:** 2025-04-24

**Authors:** Shuqi Li, Yuwang Zhang, Chenjian Liu, Xiaoran Li

**Affiliations:** Faculty of Life Science and Technology, Kunming University of Science and Technology, Kunming 650500, China; 15737288973@163.com (S.L.); m18695438757@163.com (Y.Z.)

**Keywords:** pasture, food safety, microbial source tracking, raw milk microbiota, calendar variation

## Abstract

Variations in ecological environments (including milk collection equipment and milk storage tanks in the pasture) and seasonal changes may contribute to raw milk contamination, thereby affecting food safety. The composition, structure, and relationships between raw milk and microbial communities in these environments are not well understood. In this study, 84 samples from spring and autumn in Luxian County, Yunnan Province, China, were collected for high-throughput sequencing technology. The results showed that the skin on the nipple surface and the environment (including the wiping samples of the automatic milking machine and the inner cover of the milk tank) had the greatest impact on microbial community composition in raw milk, followed by dung. In addition, microbial diversity in autumn samples was significantly higher, likely due to seasonal factors, including increased rainfall and reduced ultraviolet radiation. By analyzing the microbial community of raw milk and its environmental source, this study traced the origin of microorganisms in milk, providing insights for further exploration of the interaction between the pasture environment and raw milk microorganisms.

## 1. Introduction

Milk is consumed daily by billions worldwide due to its rich nutrient content [1]. It is considered a complete food, providing essential macro- and micronutrients for human growth and development [2,3]. The composition of raw cow’s milk encompasses a diverse range of bacterial populations, including beneficial probiotics like lactic acid bacteria, as well as pathogenic bacteria that contribute to milk spoilage and human diseases. Therefore, milk must undergo thermal treatment before it reaches consumers.

The current methods for milk thermal treatment include ultra-high temperature sterilization and pasteurization. Pasteurization plays a key role in extending the shelf life of milk and improving product quality [4,5]. A study found that consumers prefer high-temperature short-time (HTST) liquid milk for its taste and nutritional value [6]. Compared with ultra-high-temperature sterilized milk, pasteurization can maximize the retention of fresh milk nutrients and good taste. However, this may also induce milk spoilage and reduce milk quality. The proper storage temperature of pasteurized milk is vital for minimizing bacterial growth [7]. Numerous studies have demonstrated the risks associated with inadequate refrigeration, as insufficient cold storage can promote the growth of potentially harmful pathogenic microorganisms in food products, particularly in raw and pasteurized milk items [8,9]. However, in some cases, the production and transportation of pasteurized milk is difficult to maintain in low temperatures, which increases the risk of pasteurized milk spoilage [10]. Zhuang et al. found that the dominant genera in raw milk evolved from *Acinetobacter*, *Streptococcus*, *Staphylococcus*, and *Anaplasma* to *Flavobacterium*, *Pseudomonas*, and *Lactococcus* during cold storage [11]. Lan et al. found that the microbial count in milk exhibited a significant increase over a period of 16 days, even when stored at a temperature as low as 4 °C [12]. Therefore, proper control of microorganisms in raw milk, especially spoilage microbes, is an important factor for ensuring its safety and extending the shelf life of pasteurized milk.

So, where do the microorganisms in raw milk come from? Verdier et al. have proposed that the skin on the surface of the nipple is a source of microbial populations in raw milk. In contrast, Doyle et al. have suggested that herd habitat is an important driver of milk microbial community composition, while nipple preparation has a much lesser impact on raw milk microbial community [13,14]. O’Connel et al. have indicated that microbial contamination of raw milk may be related to several risk factors associated with pasture, such as unclean pasture, inadequate cleaning of milking equipment, and imperfect disinfection procedures. These factors can affect the microbial structure and composition of raw milk [15]. Consequently, the impact of the pasture environment on microbial composition in raw milk and dairy products has attracted increasing attention [16]. Numerous ecological environments together constitute the exposure environment of dairy cows, and there are a large number of microorganisms present in these environments. The transfer of microorganisms from pasture to raw milk may be influenced by various factors [17], including external contamination, such as milk containers and milk tanks, as well as cow-related sources, including mastitis occurring on the nipple surface and microbes from the intestine to the udder duct, potentially linked to forage and drinking water. Microbes can be transferred to milk through dirty udders, inappropriately sterilized milking equipment, and cows with subclinical mastitis at any stage of the milking process [18,19]. Oultram et al. used 16S rRNA gene-based high-throughput sequencing technology to compare the microbial diversity of milk samples from dairy cows with mastitis and healthy dairy cows and found that *Trueperella pyogenes*, *Streptococcus dysgalactiae*, and *Staphyloccocus aureus* had high detection rates in milk from mastitis dairy cows [20]. Makovec et al. analyzed the milk samples submitted to the Wisconsin Veterinary Diagnostic Laboratory between 1991 and 2001. The results showed that the proportion of bacteria isolated from milk samples was significantly different in the year and season, and the isolation of environmental and infectious mastitis pathogens also showed seasonal differences [21]. Additionally, sequence-based microbiota analysis was used to understand the microbial diversity of dung and nipples in order to assess the impact of transfer to milk processing plants [22,23,24]. It is very important to study the influence of dairy cows’ growth environment on milk, but so far, studies on the effects of forage, drinking water, and milk intake on the presence of microorganisms in raw milk have been one-sided, mainly focused on the comparisons between the microbial composition of healthy milk and mastitis milk samples, and there has been no complete systematic study [25,26,27].

Therefore, an in-depth study of microorganisms in the pasture environment and their impact on raw milk is essential to improve the safety and quality of raw milk and its potential impact on human health. Yunnan Province, China, is located on a plateau and does not have four distinct seasons; its annual average temperature is above 15.7 °C. Spring is dry, while autumn is rainy (the autumn rainfall accounts for more than 80% of the annual total rainfall). Ultraviolet radiation is strong in spring, with an average value of 30–40 W/m^2^ and a maximum value of 45–59 W/m^2^; in autumn, it ranges from 10 to 15 W/m^2^, with a maximum of 15–30 W/m^2^.

In this study, a total of thirty-four samples were obtained in spring (including ten environmental samples—such as those from the inner covers of the milk extractor and milk storage tank—twelve nipple samples, two raw milk samples, eight forage samples, and two drinking water samples) and fifty samples obtained in autumn (including eleven environmental samples, fourteen nipple samples, nine raw milk samples, five forage samples, six dung samples, and five biogas residue samples) for further study. This study aims to identify primary microbial contamination sources in raw milk and evaluate the impact of the pasture environment and seasonal variations on microbial diversity.

## 2. Materials and Methods

### 2.1. Sample Collection

**Figure 1:**
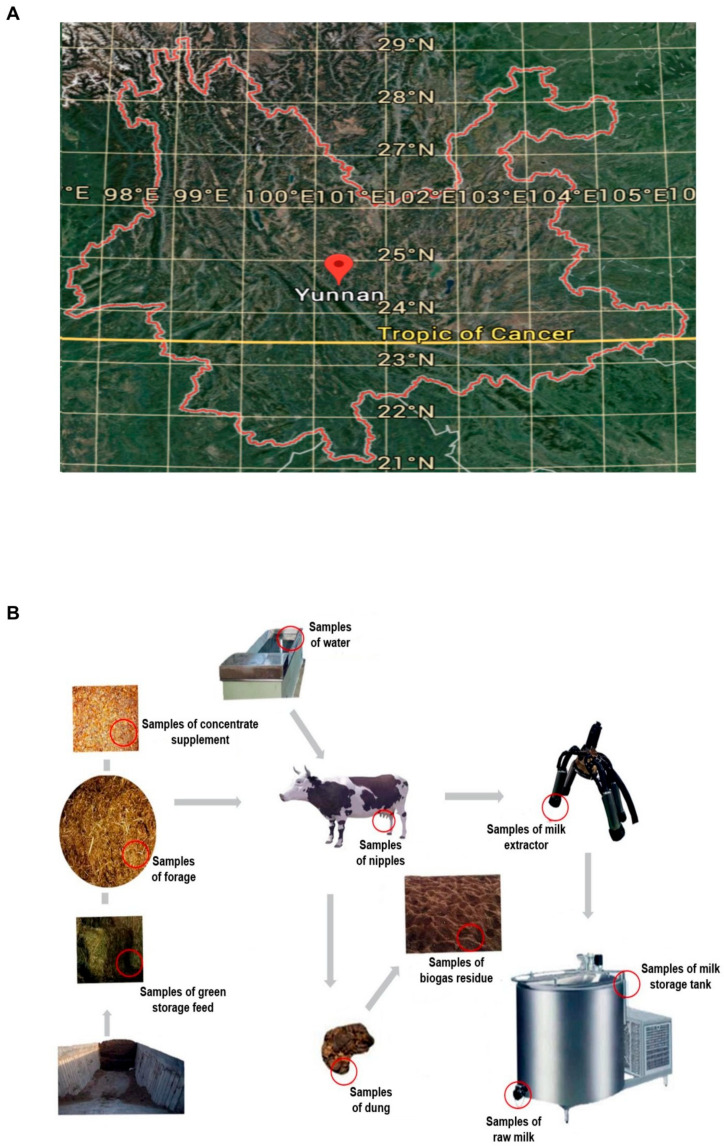
Map of Yunnan Province, China, and distribution of sampling points in pasture. (**A**) Map of Yunnan Province, China. (**B**) Distribution of sampling points for different types of samples.

The Ranch of Luxi County, Honghe Hani Autonomous Prefecture, located in Southeast Yunnan Province (Figure 1A), was selected for sampling. Yunnan is situated on a plateau with an average elevation of 2000 m, and there is no obvious distinction between the four seasons; spring is the dry season, while autumn is the rainy season, during which the pasture is located in a basin. The dairy cows in the pasture were Holstein cows aged 2–3 years and were fed a mixed diet consisting of corn silage, concentrated feed (a mixture of cornmeal, soybean meal, and crushed soybeans), and alfalfa hay.

Samples of the environment (E(S); E(A)), nipples (P(S); P(A)), raw milk (M(S); M(A)), forage (F(S); F(A)), water (W(S)), dung (S(A)), and biogas residue (B(A)) during milk extraction in the pasture were taken, respectively. The drinking water for the dairy cows was purified water from the reservoir, and the feed comprised a mixture of corn, soybean meal, distiller’s grains, protein feed, and silage (Table 1).

The ensilage was made by storing alfalfa in the ensilage pit and allowing it to ferment with microorganisms. The samples of dung included high-yielding, low-yielding, and medium-yielding dairy cow dung samples. Environmental samples were collected during dairy collection, including samples from the dairy extractor, inner cover wipe samples of the milk storage tank, and nipple wipe samples, which included high-yielding, low-yielding, and medium-yielding dairy cow nipple samples (with milk yield per cow being 18 kg; low-yielding dairy cows had a daily milk production of 18 to 35 kg, and high-yielding dairy cows produced 35 kg or more) (Figure 1B).

Wipe samples from the milk remover, milk storage tank cover, and nipples were collected with sterile cotton swabs dipped in a small amount of sterilized saline, after which the they were placed in a sterile tube and stored at −20 °C prior to DNA extraction.

### 2.2. DNA Preparation, PCR Amplification and Sequencing

Sample processing and DNA extraction were performed meticulously in a strictly controlled and sterile environment. A strict negative control was set up in the experiment, and laboratory sterile water was selected as the control to participate in the subsequent experiment and detect whether there was any pollution problem in the swabs, centrifuge tube (both the swab and centrifuge tube used in the control experiment were exposed to the sampling environment), reagents, consumables used for DNA extraction, and operation process. Microbial DNA was extracted from all samples using the QIAamp Pro Prowerfecal DNA Kit (Qiagen, Hilden, Germany) according to the manufacturer’s protocol. In addition, each sample was processed by an independent reagent group. For each sample, microbes and archaeal 16S rRNA genes were amplified with barcode primer sets 515F and 909R [28,29], including Illumina MiSeq adaptor sequences. Amplicons were purified with an ultraclean polymerase chain reaction purification kit (Mobio, Carlsbad, CA, USA). The DNA yield of the PCR product was assessed using Nanodrop 2000 (Thermoelectric Fisher, Waltham, MA, USA) and an equal amount of each sample was sequenced with the Illumina MISEQ TM system (Illumina, San Diego, CA, USA).

### 2.3. Sequence Analysis and Statistics

The analysis of quality and safety indicators of raw milk samples was completed at the Comprehensive Quality and Technical Supervision and Testing Center of Honghe Prefecture, Yunnan Province, China. All sequences were multiplexed using the barcode of each sample. Sequence processing was performed according to MiSeq SOP by combining features of Mothur v1.43.1 [30]. The SSU rRNA database sequence and classification information from SILVA (v132) can be downloaded directly from the Mothur website [31]. After aligning the sequences, chimeras were checked, and similar sequences were clustered into OTUs with a minimum recognition rate of 97%. The total sequence number > 100 OTUs in spring and autumn raw milk samples was selected for subsequent analysis. A *t*-test (two-tailed test) was used in the correlation analysis (*p* > 0.05 means no significant difference; 0.01 < *p* < 0.05 means significant difference; *p* < 0.01 indicates extremely significant difference). The distance matrix and principal coordinate analysis (PCoA) were analyzed using the Jclass method. Statistical analysis was performed with Excel 2019 and GraphPad Prism 8.

### 2.4. Nucleotide Sequence Availability

The PCR product sequencing data in this study were deposited in the Short Read Archive of NCBI under the accession number PRJNA682482.

## 3. Results

### 3.1. Quality Safety Index Analysis of Raw Milk Samples

All raw milk samples met national safety standards, with protein (3.52 g/100 g), fat (4.8 g/100 g), and microbial counts (5400 CFU/mL) within acceptable limits.

### 3.2. Variations in Microbial Abundance and Composition Across Sample Types

**Figure 2:**
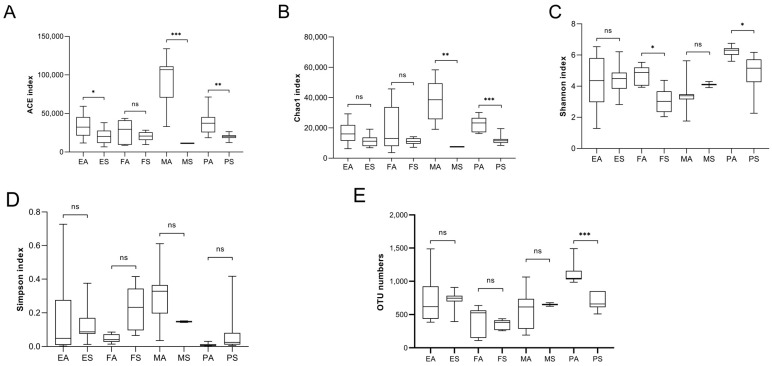
Alpha diversity between different sampling sites based on the (**A**) ACE indices, (**B**) Chao1, (**C**) Shannon, (**D**) Simpson, (**E**) OTU numbers. *t*-test, *p* values represented as ^ns^ *p*  >  0.05, * *p*  <  0.05, ** *p*  <  0.01, and *** *p*  <  0.001. ES: samples of the spring environment (including samples of the inner cover wiping of the milk extractor and milk storage tank; spring water); PS: samples of spring nipples; MS: samples of spring raw milk; FS: samples of spring forage; EA: samples of the autumn environment (including samples of the inner cover wiping of the milk extractor and milk storage tank the samples of autumn dung and biogas residue); PA: samples of autumn nipples; MA: samples of autumn raw milk; FA: samples of autumn forage.

**Figure 3:**
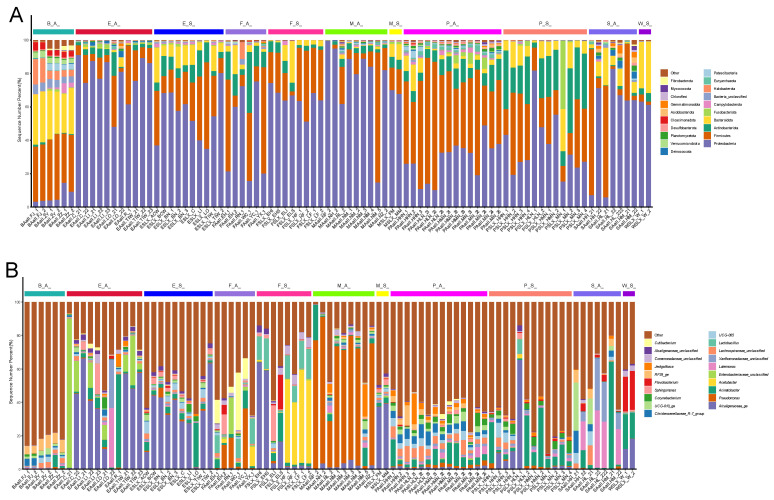
(**A**) The relative abundance of bacterial groups identified at the gate level in each pasture sample and the diversity of the most abundant bacterial groups identified at the phylum level in different groups. (**B**) The relative abundance of bacterial groups identified at the bacterial genera level in each pasture sample. Taxonomic assignment of 16S rRNA sequences was carried out with the SILVA SSU database v132 with a cutoff of 97% homology. E_S_: samples of the spring environment (including samples of the inner cover wiping of the milk extractor and milk storage tank); P_S_: samples of spring nipples; M_S_: samples of spring raw milk; F_S_: samples of spring forage; W_S_: samples of spring water; E_A_: samples of the autumn environment; P_A_: samples of autumn nipples; M_A_: samples of autumn raw milk; F_A_: samples of autumn forage; S_A_: samples of autumn dung; B_A_: samples of autumn biogas residue.

Alpha diversity can be used to determine the microbial diversity of a given sample [32]. Considering the different α-diversity indices (OTU numbers and ACE, Chao1, Shannon, and Simpson indices), significant differences were observed among most types of samples (Figure 2). Differences were found not only between different types of samples within the same season but also within the same sample types across different seasons. In general, the samples obtained in autumn exhibited higher microbial diversity, while those obtained in spring showed more uniform microbial community diversity compared to other samples. An analysis of OTUs revealed that all bacteria were distributed across 43 phyla, with Proteobacteria being the most abundant phylum (Figure 3A). There were 1231 bacterial genera at the generic level (Figure 3B). *Pseudomonas* was the most abundant genus in the autumn raw milk samples, whereas *Achromobacter* was the most abundant genus in the autumn environmental samples. *Lactobacillus* was predominantly detected in autumn samples, particularly in raw milk (M(A)), environmental wipes (E(A)), and biogas residue (B(A)). Notably, *Lactobacillus* was absent in spring raw milk but accounted for 3.2% of sequences in autumn raw milk. No significant differences were observed between spring raw milk samples, spring nipple samples, and spring environmental samples (*p* > 0.05). Similarly, there were no significant differences between autumn raw milk samples, autumn nipple samples, autumn biogas residue samples, and autumn environmental samples (*p* > 0.05, Figure 3).

### 3.3. OTU-Based Analysis

**Figure 4:**
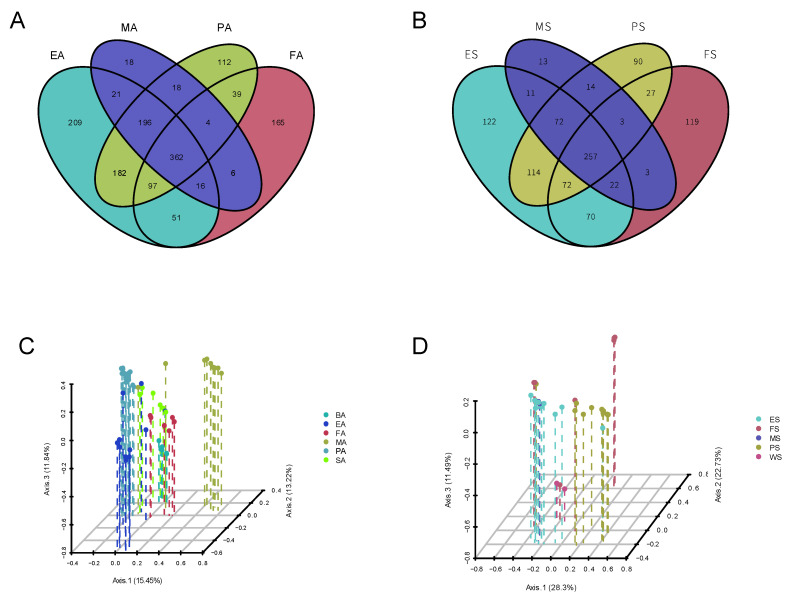
Comparison of microbiota in different samples in spring and autumn. (**A**,**B**) Venn diagram of serial numbers shared by different types of samples in different seasons. (**C**,**D**) PCoA-3D maps based on relative taxa abundance. Samples are marked with different colored group types (see legend). ES: samples of the spring environment (including samples of the inner cover wiping of the milk extractor and milk storage tank; spring water); PS: samples of spring nipples; MS: samples of spring raw milk; FS: samples of spring forage; EA: samples of the autumn environment (including samples of the inner cover wiping of the milk extractor and milk storage tank the samples of autumn dung and biogas residue); PA: samples of autumn nipples; MA: samples of autumn raw milk; FA: samples of autumn forage.

The samples selected for this study were very useful for studying the relationship between raw milk and other types of samples. Thus, principal component analysis (PCA) was performed using OTU data (Figure 4C,D). The PCoA 3D plot showed that the dung of spring environmental samples (including samples of the inner cover wiping of the milk extractor and milk storage tank), papilla samples in spring, autumn environmental samples, spring raw milk samples, spring forage samples, spring drinking water samples, autumn dung samples, and autumn biogas residue samples intersected each other without obvious clustering (Figure 4D), while the autumn raw milk samples and autumn environmental samples (including samples of the inner cover wiping of the milk extractor and milk storage tank) displayed obvious aggregation and belonged to a cluster. OTUs in raw milk samples in spring were greatly affected by the environment, nipples, forage, drinking water, dung, and biogas residue, while OTUs in raw milk samples in autumn were hardly affected by several other types (Figure 4C).

**Figure 5:**
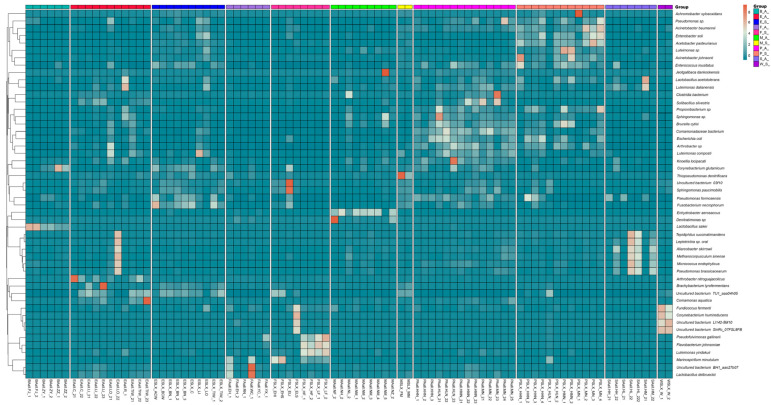
Seasonal comparison of the top 50 operational taxonomic units (OTUs) across sample types. Heat maps were generated from OTU analysis to visualize microbial abundance variations (color gradient scale: low [ultramarine] to high [orange]). ES: samples of the spring environment (including samples of the inner cover wiping of the milk extractor and milk storage tank; spring water); PS: samples of spring nipples; MS: samples of spring raw milk; FS: samples of spring forage; EA: samples of the autumn environment; PA: samples of autumn nipples; MA: samples of autumn raw milk; FA: samples of autumn forage; SA: samples of autumn dung; BA: samples of autumn biogas residue.

The 50 most abundant OTUs in the sample indicate a significant difference in the distribution of major OTUs in samples obtained in autumn and spring. Of the top 50 OTUs, OTUs mainly distributed in samples obtained in spring were *Achromobacter xylosoxidans*, *Acetobacter pasteurianus*, *Fusobacterium necrophorum*, *Glutamicibacter mysorens*, and *Flavobacterium johnsoniae*. The OTUs in autumn samples were mainly *Pseudomonas gessardii*, *Methanocorpusculum bavaricum*, *Pseudocitrobacter faecalis*, *Marine bacterium*, and *Solibacillus silvestri*. Notably, *Achromobacter xylosoxidans*, as the most abundant OTU, is prevalent in both seasons. In addition, we detected an archaea *Methanobrevibacter* among the top 50 OTUs of the sample, mainly in samples obtained in autumn, including those of feces, the environment, nipples, and raw milk. OTUs corresponding to *Lactobacillus* (e.g., *Lactobacillus delbruecki* and *Lactobacillus sakei*) were exclusively enriched in autumn samples, including raw milk, environmental wipes, and biogas residue (Figure 5).

### 3.4. Relationship Between Raw Milk and Other Types of Sample Microbial Communities

**Figure 6:**
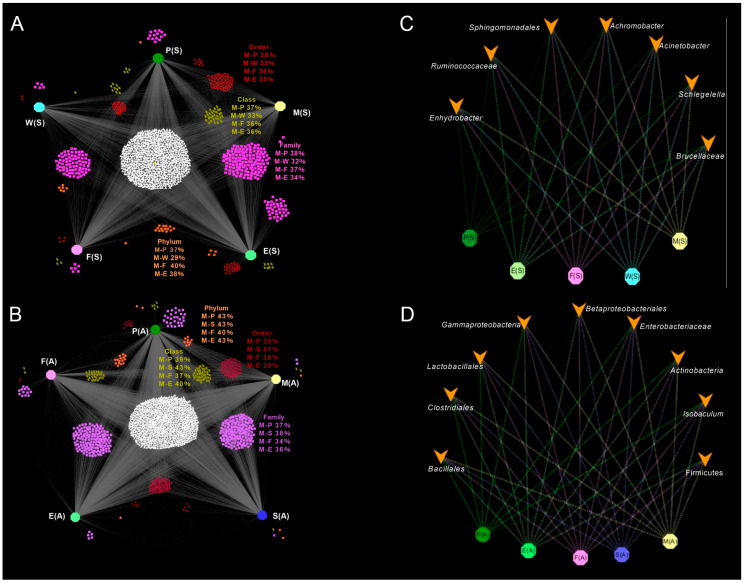
The bipartite association network of the relationship between OTUs in raw milk and other types of samples. (**A**) OTUs in spring. Octagons represent different types of samples, among which green is P(S), light yellow is M(S), light green is E(S), pink is F(S), and light blue is W(S). Scattered points of different colors represent the aggregation degrees of OTUs at different levels for each sample type. Among them, purple is at the level of Family, red is at the level of Order, light yellow is at the level of Class, and orange is at the level of Phylum. (**B**) OTUs in autumn. Octagons represent different types of samples, among which green is P(A), light yellow is M(A), light green is E(A), pink is F(A), and blue is S(A). Scattered points of different colors represent the aggregation degrees of OTUs at different levels for each sample type. Among them, purple is at the level of Family, red is at the level of Order, light yellow is at the level of Class, and orange is at the level of Phylum. (**C**) Genera with total sequence greater than 92% in raw milk samples in spring. Octagons represent different types of samples, among which green is P(S), light yellow is M(S), light green is E(S), pink is F(S), and light blue is W(S). The yellow arrows represent the bacterial categories. (**D**) Genera with total sequence greater than 91.60% in raw milk samples in autumn. Octagons represent different types of samples, among which green is P(A), light yellow is M(A), light green is E(A), pink is F(A), and blue is S(A). The yellow arrows represent the bacterial categories. E(S): samples of the spring environment (including samples of the inner cover wiping of the milk extractor and milk storage tank); P(S): samples of spring nipples; M(S): samples of spring raw milk; F(S): samples of spring forage; W(S): samples of spring water; E(A): samples of autumn environment; P(A): samples of autumn nipples; M(A): samples of autumn raw milk; F(A): samples of autumn forage; S(A): samples of autumn dung.

OTU analyses of raw milk and other types of samples were performed, expressed by the bipartite association network (Figure 6). At the phylum level, spring raw milk shared more sequence numbers with spring forage (40%), and at the class, order, and family levels, spring raw milk shared more sequence numbers with spring nipples (Figure 6A). Autumn raw milk shared the most OTUs with autumn dung (Figure 6B). Sequence numbers in raw milk samples from spring and autumn were also screened to account for more than 100 OTUs in total, in order to further analyze the relationship between several genera in raw milk and other samples. Additionally, several genera in spring raw milk samples were found to be present in other types of samples. Twelve genera in raw milk were present in both environmental and nipple samples, as well as in dung samples obtained in autumn. Compared with the autumn nipple samples and the environmental samples, biogas residue samples had fewer sequences belonging to *Achromobacter* and *Enterococcus*, and fewer *Jeotgalibaca* in autumn forage samples. Then, the raw milk samples from spring and the genera found in autumn were further screened for analysis, revealing that several genera in raw milk were present in other types of samples (Figure 6).

### 3.5. Seasonal Differences in Microbial Communities of Raw Milk and Other Samples

**Figure 7:**
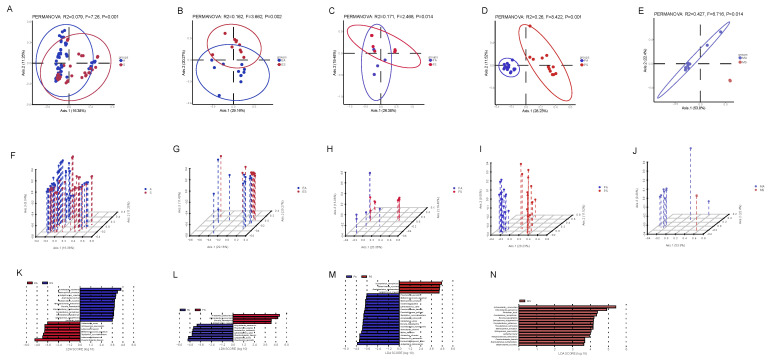
Comparison of species differences in the same types of samples during different seasons. (**A**–**E**) PCoA maps based on relative taxa abundance. (**F**–**J**) PCoA-3D maps based on relative taxa abundance. (**K**–**N**) LEfSe analysis of characteristic microorganisms in different seasons, LDA > 3.5, where red indicates characteristic microorganisms in spring. Blue shows the rich groups in the samples obtained in autumn. ES: samples of the spring environment (including samples of the inner cover wiping of the milk extractor and milk storage tank; spring water); PS: samples of spring nipples; MS: samples of spring raw milk; FS: samples of spring forage; EA: samples of the autumn environment (including samples of the inner cover wiping of the milk extractor and milk storage tank the samples of autumn dung and biogas residue); PA: samples of autumn nipples; MA: samples of autumn raw milk; FA: samples of autumn forage.

In order to explore the influence of seasons on the composition of the raw milk microbial community, we analyzed the difference in microbial community composition between samples obtained in autumn and spring. PCoA showed that there were significant differences in microbial communities among samples from different seasons (Figure 7A–G). Given the statistical differences in α and β diversity between samples obtained in autumn and spring, LEfSe analysis was performed on the first 100 OTUs to determine bacterial markers for each sample in both seasons. Of particular interest is the fact that seventeen different species were found in raw milk, and interestingly, all of these 17 species were found in spring raw milk, but not in autumn raw milk. At the same time, of these 17 species, *Fusobacterium necrophorum*, *Glutamicibacter mysorens*, and *Brachybacterium tyrofermentans* were enriched in the spring environment and spring papillae, *Brucella haematophila* was enriched in the spring environment and spring feed, and *Sphingomonas oligophenolica*, *Corynebacterium glutamicum*, *Marinospirillum minutulum*, *Janibacter hoylei*, *Halopseudomonas formosensis*, *Enhydrobacter aerosaccus*, and *Sphingomonas paucimobilis* were enriched in the spring environment. This also showed that the microbes in raw milk were highly correlated with other samples.

## 4. Discussion

It is of great significance to study the composition of and variation in microbial communities in raw milk for the prevention of food-borne pathogenic microbial contamination. This study evaluated how environmental factors (e.g., milking equipment, nipple surfaces) and seasonal changes influence raw milk microbiota. The findings underscore the need for stringent hygiene practices and offer insights into extending the shelf life of pasteurized milk.

This study highlights the critical role of environmental and seasonal factors in shaping raw milk microbiota. The higher microbial diversity observed in autumn underscores the influence of increased rainfall and reduced UV radiation, which may facilitate microbial transfer from the environment to milk. The prominent contribution of nipples and milking equipment emphasizes the need for stringent hygiene practices to minimize contamination. These findings align with previous research demonstrating the impact of environmental conditions on milk safety. Future studies should explore intervention strategies, such as optimized cleaning protocols and environmental modifications, to enhance milk quality and safety. The transfer of microorganisms from pasture to raw milk may be affected by a variety of factors [17], such as unclean pasture, inadequate cleaning of milking equipment, and imperfect disinfection procedures. Additionally, the cow’s own sources, including mastitis occurring on the nipple surface and intestinal-udder duct transfers, as well as forage and drinking water, may also play a role. These may affect the microbial structure composition of raw milk. Falardeau et al. observed 37 core OTUs in cheese, 32 of which are shared with dairy farms [33]. Regasa et al. have proposed that the prevalence of Staphylococcus aureus was “15.3% from udder milk, 25%, 20%, and 10% from milkers’ hand, milking bucket, and drying towel swab, respectively” [34]. Orwa et al. found that effective udder cleaning and high personal hygiene by hand milkers may reduce microbial contamination risk in milk production. This was determined by counting live bacteria, coliform, thermophilic, and psychrophilic bacteria in udders, hands, milking containers, filling containers, and water sources [35]. In this study, the environment (including samples of the inner cover wiping of the milk extractor and milk storage tank) has a great impact on the structure composition of microorganisms in raw milk, so it can also be said that the cleanliness of milking equipment and the milk storage tank has a great impact on the structure composition of microorganisms in raw milk. The use of cold or hot water, and whether detergent is used, will impact the cleaning process. Cold water is a potential source of microorganisms in raw milk, especially when disinfection processes are inadequate [36,37]. Some bacterial species, such as *Enterococcus*, *Lactobacillus*, and some *Corynebacterium*, can survive for only 20 min at 60 °C [38]. As a result, fewer microbial communities were found in milk extractors and tanks cleaned with hot water for extended periods compared to those cleaned with cold water. Studies have shown that the use of detergents and hot water to clean milk extractors and milk tanks can significantly reduces the number of microorganisms in raw milk [39,40]. This highlights the critical role of milking equipment cleanliness in reducing contamination risks. Dairy farms must implement rigorous cleaning protocols using hot water and detergents to limit microbial contamination. Additionally, monitoring environmental hygiene can be a cost-effective strategy to improve milk safety and extend its shelf life.

A large number of *Achromobacter* sequences were found in autumn and spring samples. However, only a small number were detected in nipples, raw milk, spring drinking water, and spring forage samples. *Achromobacter* is a ubiquitous environmental organism and Gram-negative microbe, widely existing in soil, water, and other environments [41]. *Achromobacteria* may become opportunistic pathogens in certain conditions, such as cystic fibrosis, hematologic and solid organ malignancies, renal failure, and certain immune deficiencies [42]. It is possible that insects with nematodes have been attached to the nipple surfaces of dairy cows. During milking, colorless microbes on the nipple surface enter the raw milk, causing contamination. Once the raw milk enters the tank, the nutrient-rich medium supports rapid microbial growth [43]. It is possible that such insects are seasonal and therefore do not exist in samples obtained in spring. The abundance of *Achromobacter* suggests that the autumn environment, including milk collectors and the inner cover wipes of milk tanks, as well as autumn nipples, significantly influence the microbial community structure in autumn raw milk.

This analysis showed that seasons have a great influence on the microbial community structural composition of each sample. Yunnan Province does not have four distinct seasons; spring is dry, while autumn is rainy. Consequently, the rainfall gradient and UV intensity significantly influenced microbial community composition in various samples. For instance, intestinal-related genera like *Corynebacterium*, *Ruminococcus*, *Enterococcus*, and *Achromobacter* were more prevalent in autumn raw milk samples than in spring samples [44,45]. These results indicate that autumn samples have greater microbial diversity and more intestinal microorganisms compared to spring samples. Additionally, autumn raw milk exhibits higher microbial diversity. Variations in dairy cows or their exposure environments can influence microbial community changes in samples. The observed differences in microbiota may result from rainfall gradients and UV intensity. However, this correlation requires further comprehensive investigation. Regardless of the season, the nipple surface and environment, including milk extractor inner cover wipes and milk storage tanks, are the primary contributors to raw milk microbial communities, followed by dung. This is consistent with a previous study that proposed that the skin on the nipple surface is the source of microbial populations in raw milk [46].

The observed reduction in microbial diversity during spring, characterized by high UV radiation (30–40 W/m^2^), suggests potential UV-induced microbial suppression. UV radiation is known to damage microbial DNA and inhibit replication, selecting taxa with UV-resistant traits such as pigmented bacteria (e.g., *Deinococcus*) or those capable of forming biofilms [47]. Although *Achromobacter* dominated in both seasons, its persistence under high UV conditions may be attributed to biofilm formation, as reported in soil and water systems [48]. This adaptation highlights the need to target biofilm-forming microbes during equipment cleaning. The seasonal UV gradient in Yunnan underscores the importance of tailored hygiene protocols. For instance, UV-resistant pathogens (e.g., *Achromobacter*) may require enhanced disinfection during spring. Integrating UV-C light into milking equipment sanitation could complement traditional methods, as UV-C effectively inactivates biofilm-associated bacteria. Additionally, dairy farms could monitor UV forecasts to adjust cleaning frequency, mitigating contamination risks during high-radiation periods.

From a dairy perspective, *Lactobacillus* ferments raw milk, producing lactic acid and antibacterial substances. This inhibits pathogenic bacteria growth, enhancing dairy product shelf life and safety. *Lactobacillus*, detected predominantly in autumn raw milk and environmental samples (Figure 3B), ferments raw milk by producing lactic acid and antibacterial substances. This metabolic activity inhibits pathogenic bacteria growth, thereby enhancing dairy product shelf life and safety. Additionally, *Lactobacillus* contributes to flavor compound synthesis, which was corroborated by its seasonal prevalence in autumn samples under high-humidity conditions [49,50]. *Ruminococcus* is a prevalent member of gut microbiota within the Firmicutes division. It is a strict anaerobic, Gram-positive coccus [46,51,52]. *Ruminococcus* was found in large quantities in spring samples, including feces, biogas residue, environmental samples (such as milk extractor inner cover wipes and milk storage tanks), and nipple samples. The abundance of *Ruminococcus* DNA may stem from biogas residue, made from dried feces, being used as dairy cow bedding. This likely transfers *Ruminococcus* DNA to the nipple surfaces. During milking, milk comes into contact with environmental samples, such as the inner cover wipes of milk extractors and milk storage tanks. *Corynebacterium* has also been identified as a pathogen associated with cow mastitis and is often described as infectious [20]. *Corynebacterium* was found in numerous nipple samples, as well as in raw milk and environmental samples, including milk extractor inner cover wipes and milk storage tanks. Although *Arcobacter* is associated with reproductive disorders and mastitis in livestock, *Arcobacter* has been isolated from milk and cow manure [53,54]. *Arcobacter* has also been isolated from cows with outbreaks of mastitis, as well as from healthy cows [55,56]. The large amount of DNA of *Ruminococcus*, *Corynebacterium*, and *Arcobacter* reflect the influence of skin on the structure of the microbial community in raw milk.

This study identifies nipples and milking equipment as key drivers of microbial contamination in raw milk. Additionally, rainfall gradients and UV intensity in the cow exposure environment significantly impact microbial composition. However, this relationship requires further comprehensive investigation [44,45]. Every result contributes to understanding microbial contamination dynamics in raw milk. From environmental hygiene to seasonal adjustments, the study emphasizes actionable steps that can be taken by dairy farmers, policymakers, and researchers to improve milk quality and safety. These findings also open avenues for developing microbial-based quality assurance tools and exploring the interactions between climate and microbiota.

## 5. Conclusions

This study systematically elucidates the structural and compositional dynamics of microbial communities in raw milk, demonstrating that environmental factors—notably the sanitation status of milking equipment and teat surfaces—play a pivotal role in shaping microbial quality. The findings emphasize the critical necessity of implementing rigorous hygienic protocols across dairy farming operations to mitigate contamination risks. Furthermore, seasonal variations, driven by rainfall gradients and ultraviolet radiation intensity, were identified as key determinants of microbial diversity, with autumn exhibiting markedly richer microbial profiles compared to spring. The detection of opportunistic pathogens such as *Achromobacter* and *Corynebacterium* underscores the urgency of optimizing cleaning and disinfection strategies to safeguard milk safety. Future investigations should prioritize the development of innovative disinfection technologies and elucidate microbial transfer pathways to enhance raw milk quality and dairy product safety. These insights not only advance sustainable dairy management practices but also establish a scientific framework for extending the shelf life of pasteurized milk, thereby fostering economic and public health benefits for both producers and consumers.

## Figures and Tables

**Table 1:** Experimental design and sample collection summary.

Season	Sample Type	Abbreviation	Number of Samples	Description
Spring	Environment	E(S)	10	Inner cover wipes of the milking extractor and milk storage tank
	Teat surfaces	P(S)	12	Swabs from high-, medium-, and low-yielding cows (daily milk ≥ 35 kg, 18–35 kg)
	Raw milk	M(S)	2	Collected during milking
	Forage	F(S)	8	Corn silage and concentrated feed (cornmeal and soybean meal)
	Drinking water	W(S)	2	Purified reservoir water
Autumn	Environment	E(A)	11	Inner cover wipes of the milking extractor and milk storage tank
	Teat surfaces	P(A)	14	Swabs from high-, medium-, and low-yielding cows
	Raw milk	M(A)	9	Collected during milking
	Forage	F(A)	5	Alfalfa hay and silage
	Animal feces	S(A)	6	Feces from high-, medium-, and low-yielding cows
	Biogas residue	B(A)	5	Dried residues from biogas production (used as bedding material)

## Data Availability

The original contributions presented in the study are included in the article, further inquiries can be directed to the corresponding authors.
